# World Lung Day 2025—Healthy Lungs, Healthy Life

**DOI:** 10.1111/resp.70121

**Published:** 2025-09-21

**Authors:** Kwun M. Fong, David C. L. Lam, Yoshinori Hasegawa, Suga Konno, Chul‐Gyu Yoo

**Affiliations:** ^1^ The Prince Charles Hospital and Faculty of Medicine The University of Queensland Brisbane Queensland Australia; ^2^ School of Clinical Medicine University of Hong Kong Hong Kong SAR China; ^3^ Nagoya University Graduate School of Medicine Nagoya Japan; ^4^ Asian Pacific Society of Respirology Tokyo Japan; ^5^ Department of Internal Medicine Seoul National University College of Medicine Seoul Korea

**Keywords:** asthma, COPD, lung cancer, respiratory infections, tuberculosis

The Asian Pacific Society of Respirology (APSR; https://apsr.org/) is a proud member of The Forum of International Respiratory Societies (FIRS), the peak global organisation working to improve lung health together with the American College of Chest Physicians (CHEST), American Thoracic Society (ATS), Asociación Latinoamericana De Tórax (ALAT), European Respiratory Society (ERS), International Union Against Tuberculosis and Lung Diseases (The Union), Pan African Thoracic Society (PATS), Global Initiative for Chronic Obstructive Lung Disease (GOLD), and Global Initiative for Asthma (GINA). FIRS's ~100,000‐strong membership is focused on the organisation's mission of promoting global equity in the prevention, diagnosis and treatment of respiratory disease, to improve lung health—through their member organisations, through advocacy and by working with the World Health Organization (WHO) and other global organisations (https://firsnet.org/about/).

World Lung Day (WLD) in 2025 will have seen nearly a decade of progress with key lung health initiatives since its launch at the 2016 FIRS Kyoto Assembly meeting (Figure 1) hosted by then FIRS President, Prof. Michiaki Mishima. At that time, FIRS recognised the need for new initiatives to bring people and lung health communities together to help meet the United Nations Sustainable Development Goals (SDGs) especially given the uneven rate of global industrialisation amplifying lung health gaps for the most vulnerable (age extremes, economic disadvantage, social deprivation, poor living conditions, tobacco smoking, pollution and environmental exposures).

**FIGURE 1:**
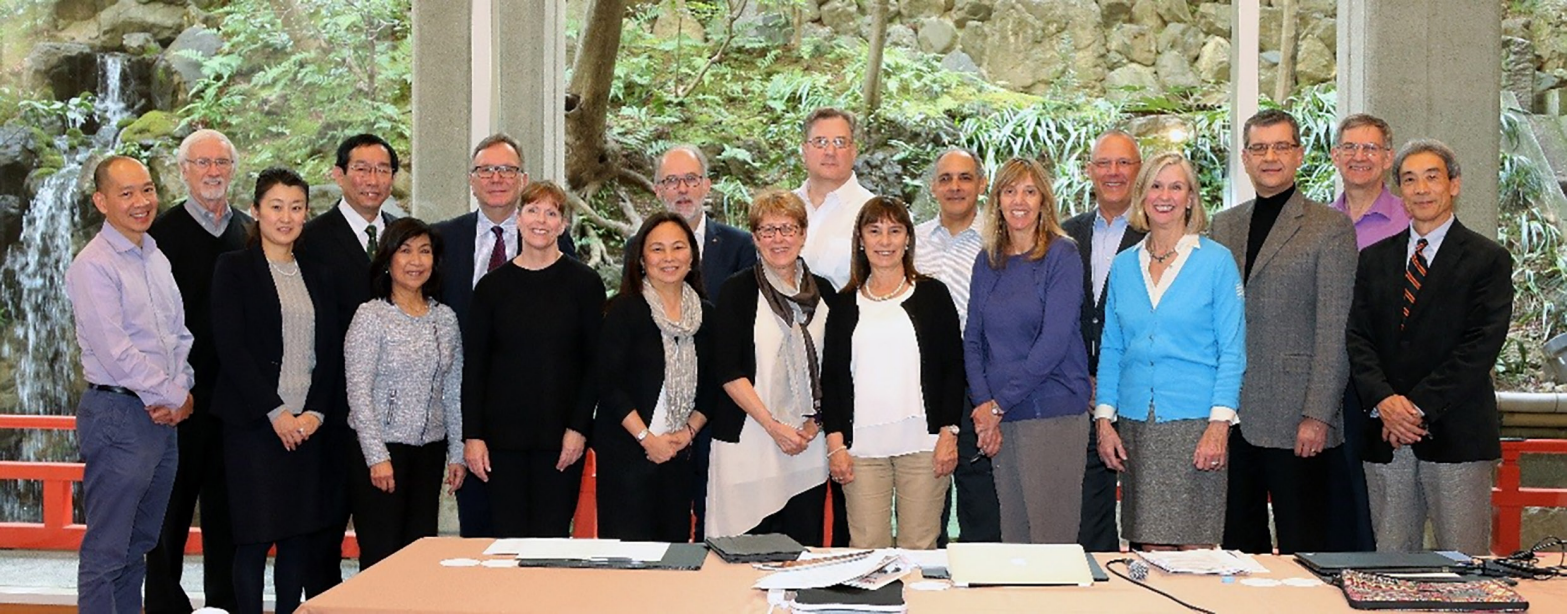
FIRS Meeting, Kyoto, Japan 4–5 February 2016. Pictured: M. Mishima (APSR), A. Casas, V. Lopez Varela (ALAT); D. Marciniuk, B. Phillips, P. Markowski (CHEST); K. Fong, Y. Yamanaka, R. Kishigami (APSR); T. Ferkol, S. Crane, F. du Melle (ATS); G. Joos (ERS); E. Jane Carter, D. Schraufnagel, P. Fujiwara (The Union); H. Zar (PATS), N. Billo (FIRS Director) FIRS Secretariat: B. Sax (ERS).

World Lung Day is observed on September 25th each year, as an inclusive global campaign to raise awareness about lung health and the urgent need to reduce the burden of respiratory diseases. FIRS and its growing list of WLD partners (https://firsnet.org/world‐lung‐day‐2025/partners/) highlight gaps and opportunities for policy makers and health care stakeholders at all levels. In a world marked by challenges from political differences and conflicts, environmental challenges and socioeconomic disparities, FIRS' support and investment in lung health remain crucial in view of all these competing issues impacting on lung health.

WLD draws attention to lung diseases that cause much health burden. The lung is the internal organ most vulnerable to infection and injury from the external environment because of constant exposure to particles, chemicals, and infectious organisms in ambient air. Globally, at least 2 billion people are exposed to the toxic smoke of biomass fuel combustion, typically burned inefficiently with poorly ventilated indoor stoves or fireplaces. It was estimated that 1 billion people are exposed to outdoor air pollution, and 1 billion are exposed to tobacco smoke [1, 2]. While recent attention has focused on acute respiratory conditions related to respiratory infections such as COVID‐19, chronic respiratory diseases also remain a priority contributing to the burden of non‐communicable diseases (NCDs) which accounts for approximately 70% of global deaths, disproportionately affecting the poor and people in low‐ and middle‐income countries (LMICs) [3, 4].

Five of these diseases (the Big 5) are among the most common causes of severe respiratory illness and death worldwide—COPD, asthma, acute lower tract infections, tuberculosis, and lung cancer [2].

- More than 65 million people suffer from COPD, the third leading cause of death worldwide–and the numbers are increasing.
- Asthma is the most common chronic disease of childhood, affecting 14% of children and its prevalence in children is rising.
- Tuberculosis is the most fatal infectious disease, with 10.4 million cases and 1.4 million deaths annually.
- Lung cancer is the most common cause of neoplasm‐related death in the world–and the numbers are growing.
- Pneumonia is the top cause of death for decades and is the leading cause of death in children under the age of 5.

Other respiratory conditions are also important for the Asian Pacific region:

- Sleep apnoea affects more than 100 million people—up to 10% of adults in some populations.
- Occupational lung disease affects more than 50 million people and workers continue to breathe in sickening mineral dusts, bioaerosols, and fumes.
- Pulmonary hypertension occurs in 1% of the world's population and 10% of those over 65 years of age. Pulmonary embolism has an incidence reported to be at 6 to 20 per hundred thousand–but this may be grossly underestimated.

The ‘Big 5’ causes most of the burden from lung diseases in the context of environmental issues embracing both outdoor and indoor air pollution (including tobacco smoking) and represents a bundle of major challenges that APSR is addressing with like‐minded individuals and organisations. This is entirely in keeping with its objectives to encourage research, to improve clinical practice through training, to increase awareness of health problems and to promote the exchange of knowledge among respirologists in the Asia‐Pacific region and beyond (https://apsr.org/about/apsr/). The APSR combines talents and resources from collaborators to make a difference to respiratory health, which aligns with the objectives of the Big 5 workshop held in conjunction with the annual APSR Congresses (https://apsr.org/events/congress/).

APSR members are also members of major local respiratory societies in the respective region, including many low and middle income (LMIC) countries where resource constraints are prominent yet the burden of the Big 5 is disproportionately high [5]. Nonetheless, APSR is optimistic that solutions can be found to overcome challenges in their prevention, diagnosis, and management underpinned by science, evidence, and action, with the ‘glue’ being awareness, scalable collaborative actions, and resourcing as opportuned by WLD Calls for Action.

The Big 5 workshop (https://apsr.org/education‐science/big5‐lung‐diseases/) focuses attention on implementation of effective primary care respiratory interventions that make a difference at the individual and community levels. The first ‘Towards regional progress: APSR 2022 Big Five Lung Diseases Workshop’ was jointly organised and greatly benefited from a Union APR partnership. Representatives nominated by WHO SEARO and guidance from WHO WPRO contributed to a highly productive workshop, with a specific focus on primary care models tailored to low‐ and middle‐income countries. Summaries of these discussions were published as a freely accessible tool [6]. As part of the APSR's longer term commitment to addressing the Big 5, workshop toolkits were developed and are also freely available thanks to the generosity of the Investigators (Table 1; https://apsr.org/education‐science/big5‐lung‐diseases/). To date, these have been downloaded over 140 times by healthcare workers from hospitals, clinics, NGOs, and community health organisations in low‐ to upper‐middle‐income countries. APSR encourages dissemination and sharing of impactful clinical initiatives for LMICs in the AP region and beyond. Building on this success, APSR invites interested people to consider participation in the 2nd Big 5 Lung Disease Workshop on **10 November 2025**, during the APSR Congress in Manila (https://2025.apsr.org/programme/workshop/information).

**TABLE 1:** Toolkit links from the Big Five Lung Diseases Workshop.

1. Activating primary care COPD patients with multimorbidity (APCOM) programme
2. All‐hands on deck approach to Covid‐19 vaccination—Oriental Mindoro
3. Easy asthma clinic network
4. Peak inspiratory flow rate guided inhalation therapy
5. Improved clinical efficacy of indeterminant pulmonary nodules through lung cancer biomarker panel risk model and optimized diagnostics pathway for lung cancer
6. Community respiratory centre/best practice model for the management of Big‐5 respiratory illness
7. Ubo patrol: a model of primary care for the Big 5 lung diseases in Dagupan City, Province of Pangasinan, Philippines
8. Co‐care for DM‐latent infection of TB (CO4DLIT)
9. The big five lung diseases in Ho Chi Minh City

There are compelling reasons to enable WLD to draw attention and to spur efforts to contribute to healthier lungs for the global population:

- **Raising awareness**: Perhaps under‐recognised in the general public, lung diseases are a leading cause of death and disability globally.
- **Prevention**: WLD bring awareness to the importance of preventive measures such as reducing air pollution, linking to resources for self‐care including promoting smoking cessation, avoiding occupational exposure, indoor and outdoor air pollution, and vaccinations to maintain good lung health.
- **Access to care**: As a global effort, WLD showcases variation and heterogeneity in lung health services, health care access and equity. Such disparities inhibit quality respiratory care, particularly in low‐ and middle‐income countries. WLD emphasises the need for equitable access for all to effective health technologies.
- **Climate change and air quality**: WLD illustrates the effect of climate change and environment on lung health through increased air pollution and the spread of respiratory infections.
- **Advocating for research**: Scientific evidence, clinical trials and research are the foundation stones for improvement in health prevention, diagnosis and treatments. World Lung Day encourages research to identify gaps and develop better diagnostic tools, treatments, and preventative strategies to transform lung diseases.
- **Worldwide empowerment and collaboration**: World Lung Day fosters collaboration among respiratory societies, healthcare professionals, policymakers, non‐governmental organisations, and individuals to focus on the global lung health crisis.

This year's WLD theme is ‘Healthy Lungs, Healthy Life’ (https://firsnet.org/world‐lung‐day‐2025/). Lung health, while vital to human health, has not been front of mind for many for too long! To achieve lung health in the global conscience, we need to be at the table and heard through all channels for political will to enable changes based on solid research and clinical evidence. APSR strongly supports World Lung Day, 25th September, as the day to reflect, share with community and advocate for better lung health. Please help spread the work, with a comprehensive toolkit available including practical graphics on lung health care freely available to disseminate on social media (https://firsnet.org/world‐lung‐day‐2025/). With 5 years left to achieve the Sustainable Development Goals and the 4th High‐level Meeting of the United Nations General Assembly on the Prevention and Control of NCDs due in September 2025, now is the time to celebrate and amplify WLD!

## Conflicts of Interest

The authors declare no conflicts of interest.
